# Supplementary material for: Prevalence and risk factors of hypertension among adults: A community based study in Addis Ababa, Ethiopia
Source: PLoS One. 2021 Apr 1;16(4):e0248934. doi: 10.1371/journal.pone.0248934 (PMC8016337; doi:10.1371/journal.pone.0248934)
Supplement: S1 Annex — (PDF) [file pone.0248934.s002.pdf]

QUESTIONNAIR ID /

Annex I: English version questionnaires

ADDIS ABABA UNIVERSITY
COLLEGE OF HEALTH SCIENCES
SCHOOL OF PUBLIC HEALTH

Questionnaire designed to study Epidemiology of Common Non Communicable Diseases (Hypertension, diabetes mellitus and Dyslipidemia) among Adults in Addis Ababa, Ethiopia: A Community Based Study

Survey information

**Location and date**	**Response**	**Code**	**Skip to**
Cluster/ village ID	---------------	I1	
Sub city name	....	I2	
Cluster/ village name	--------------	I3	
Interviewer Name and ID	--------------	I4	
Date of completion of the questionnaires	dd/mm/year-----,--------,----------	I5	
**Consent, interview language and name**	**Response**	**Code**	
Consent has been read and obtained	1, yes 2, No if no , end	I6	
Interview language	Amharic	I7	
Time of interview (24 hour clock)	Hours-------, mins------	I8	
Participant code number	---------------------------------	I9	
Name of participant	---------------------------------	I10	
Contact phone number where possible	--------------------------------	I11	
**Step 1 Demographic information**			
**CORE: Demographic information**			
**Question**	**Response**	**Code**	**Skip to**

QUESTIONNAIR ID /

Sex	Male --------------1<br>Female------------2	C1	
What is your date of birth?	dd/mm/yr<br>Don't know 77	C2	If known➔<br>C4
How old are you?	……… in years	C3	
Where was your birth place?	Rural ---------------1<br>Urban---------------2<br>I don't know-------3	C4	
For how many years did you live in Addis Ababa?	------------ in months<br>-------------in years<br>Born in Addis Ababa-----1	C5	
In total, how many years have you spent at school and in full-time study (excluding pre-school)?	……… in years	C6	
**Expanded: Demographic information**			
Have you ever attended school?	Yes----------------------1<br>No ----------------------2	C7	
What is the highest level of school you attended:	Primary (1-8)--------------1<br>Secondary (9&10)------- 2<br>Preparatory(11 &12)--- -3<br>Technical/Vocational ---4<br>University -------- 5<br>Refused---------------88	C8	
What is your Ethnic group?	Amhara-----------------------------1<br>Oromo -----------------------------2<br>Gurage -----------------------------3<br>Tigre -------------------------------4<br>Other---------- -------------------- 5<br>Other, specify----------------------------	C9	

QUESTIONNAIR ID /

What is your marital status?	Single never married-------------1<br>Currently married--------------- 2<br>Separated-------------------------3<br>Divorced---------------------------4<br>Widowed--------------------------5<br>Refused ---------------------------88	C10	
What is your religion?	Orthodox -------------------------1<br>Muslim------------------------- 2<br>Protestant--------------------------3<br>Catholic----------------------------4<br>Other--------------------------------5<br>Other, specify-------------------------------	C11	
Which of the following best describes your main work status over the past 12 months?	Government employee-------------1<br>Non government employee--------2<br>Self employed-----------------------3<br>Student-------------------------------4<br>Housewife--------------------------5<br>Daily laborer--------------------6<br>Merchant-------------------------7<br>Unemployed(able to work)-----8<br>Unemployed(unable to work)-----9<br>Retired-------------------------- 10<br>Other--------------------------------11<br>Other, specify------------------------	C12	
How many people older than 18 years, including yourself, live in your household?	Number of people.............	C13	
How many people live in your household including yourself?	Number of people--------------------	C14	

Can you tell me the monthly income of the household?	...............................birr-------1<br>Didn't have any income ------2<br>Refused ----------------------- 88			C15	
**Step 1 Behavioral measurements**					
**CORE: Tobacco use**					
Now I am going to ask you some questions about tobacco use					
**Question**	**Response**			**Code**	**Skip to**
Do you currently smoke any tobacco products, such as cigarettes, cigars, Gaya or pipes?	Yes------------- 1<br>No -------------- 2			T1	If no, ➔ T8
Do you currently smoke tobacco products daily?	Yes ------------- 1<br>No---------------- 2			T2	
How old were you when you first started smoking?	Age in years ---------------------<br>Don't know------------------- 77			T3	If known, ➔ T5a/T5aw
Do you remember how long ago it was?<br>(Record only one, not all 3)	In years --------			T4a	If known, ➔ T5a/T5aw
	Or in months-----------------			T4b	If known ➔ T5a/T5aw
	Or in weeks-----------------------			T4c	If known ➔ T5a/T5aw
On average, how many of the following products do you smoke each day/ week?<br>Don't know 7777		Daily	Weekly		
	Manufactured cigarettes			T5a/Tsaw	
	Hand rolled cigarettes			T5b/Tsbw	
	Pipes full of tobacco			T5c/Tscw	

	Cigars, cheroots, cigarillos			T5d/Tsdw	
	Number of Shisha sessions			T5e/Tsew	
	Other			T5f/Tsfw	
	If other, go to T5 other, else go to T6				
During the past 12 months, have you tried to stop smoking?	Yes ------------- 1<br>No ------------- 2			T6	
During any visit to a doctor or other health worker in the past 12 months, were you advised to quit smoking tobacco?	Yes--- 1			T7	If T2=yes, ➔ T12,<br>if T2=No, ➔ T9
	No-- 2				If T2=yes, ➔ T12,<br>if T2=No, ➔ T9
	No visit during the past 12 months---- 3				If T2=yes,, go to T12, ➔<br>if T2=No, ➔

			T9
In the past, did you ever smoked any tobacco products?	Yes----------------------- 1<br>No ----------------------- 2	T8	If no, ➔ T 12
In the past, did you ever smoked daily?	Yes---------------------- 1<br>No----------------------- 2	T9	If T1=yes, ➔ T12, else ➔ T10<br>If T1=yes, ➔ T12, else ➔ T10
**EXPANDED: TOBACCO USE**			
**Question**	**Response**	**Code**	**Skip to**
How old were you when you stopped smoking?	Age (years)----------------------<br>Don't know -----------------77	T10	If known, ➔ T12
How long ago did you stop smoking?<br>(Record only one answer)	Years ago -----------<br>Or months ago-----<br>Or weeks age------<br>Don't know ---77	T11a	If known, ➔ T12
		T11b	If known, ➔ T12
		T11c	
Do you currently use any smokeless tobacco products such as (snuff, chewing tobacco, betel)?	Yes 1<br>No 2	T 12	If no, ➔ T15

Do you currently use smokeless tobacco products daily?	Yes 1<br>No 2			T13	If no, ➔ T14aw
On average, how many times a day/ week you use<br>(If less than daily, record weekly)<br>Don't know 777		Daily	Weekly		
	Snuff, by mouth			T14a/<br>T14aw	
	Snuff, by nose			T14b/<br>T14bw	
	Chewing tobacco			T14c/<br>T14cw	
	Other *If Other, go to T14other, if T13=No, go to T16, else go to T17*			T14d/<br>T14dw	
	Other(please specify):			T14other/<br>T14otherw	*If T13=No,* ➔ *T16, else* ➔ *T17*
In the past, did you ever use smokeless tobacco products such as (snuff, chewing tobacco, or betel)?	Yes-------------- 1<br>No ----------------2			T15	If no, ➔ T17
In the past, did you ever use smokeless tobacco products such as [snuff, chewing tobacco, or betel]daily?	Yes ---------- 1<br>No ---------- 2			T16	
During the past 30 days, did someone smoke in your home? For never smoke only)	Yes------- 1<br>No------- 2			T17	

During the past 30 days, did someone smoke in closed areas in your workplace (in the building, in a work area or a specific office)?	Yes---------------------------- 1<br>No---------------------------- 2<br>Don't work in a closed area-- 3	T18	
Are you currently chew Khat?	*Yes* --------------------- *1*<br>*No* -------------------- *2*<br>*Refused*------------- *88*	T20	If no ➡ T26
During the last 30 days, on average how many days did you chew Khat?	Daily ---------------------1<br>Weekly -------------------2<br>Less than a month------3<br>Monthly------------------4<br>Other----------------------5<br>Other, specify---------------	T21	
How many **Zurbas/"esire"** do you chew on **one** of those days?	---------- Zurbas/"esire"	T21a	
How old were you when you first started chewing Khat?	Age in years----------<br>I don't know-------77	T22	
Do you remember how long ago it was, you have been started chewing Khat?	Weeks --------<br>Months ---------<br>Years ----------	T23	
In the past, did you ever chew Khat? (For currently non Khat chewer)	Yes-------------------------1<br>No--------------------------2<br>No response----------------88	T26	If no ➡ A1

QUESTIONNAIR ID /

How old were you when you stopped chewing Khat?	Age in years--------	T27	
How long ago did you stop chewing Khat?	Weeks --------<br>Months ---------<br>Years ----------	T28	
**CORE: Alcohol Consumption**			
The next questions ask about the consumption of alcohol			
**Question**	**Response**	**Code**	**Skip to**
Have you ever consumed any alcohol such as Beer, Wine, Spirits, Whisky, etc.?	Yes 1<br>No 2	A1	If no, ➔ A16
Have you consumed any alcohol within the past 12 months?	Yes 1<br>No 2	A2	If no, ➔ A4
Have you stopped drinking due to health reasons, such as a negative impact on your health or on the advice of your doctor or other health worker?	Yes 1<br>No 2	A3	If yes, ➔ A16<br>If no, ➔ A16
During the past 12 months, **how frequently** have you had at least one standard alcoholic drink?	Daily 1<br>5-6 days per week 2<br>3-4 days per week 3<br>1-2 days per week 4<br>1-3 days per month 5<br>Less than once a month 6	A4	
Have you consumed any alcohol within the **past 30 days**?	Yes 1<br>No 2	A5	If no, ➔ A13
During the past 30 days, on how many **occasions** did you have at least one standard alcoholic drink?	Number ---------------------<br>Don't know ----------------77	A6	

During the past 30 days, when you drank alcohol, how many **standard drinks on average** did you have during one drinking occasion?	Number -----------------<br>Don't know ---------------77	A7	
During the past 30 days, what was the **largest number** of standard drinks you had on a single occasion, counting all types of alcoholic drinks together?	Largest number--------------<br>Don't know ------------------77	A8	
During the past 30 days, how many times did you have **six or more** standard drinks in a single drinking occasion?	Number of times ------------------<br>Don't know ------------------------77	A9	
For men: five or more	Number of times---------<br>Don't know --------------77	A9a	
For women: four or more	Number of times --------------------<br>Don't know -----------------------77	A9b	
During each of the **past 7 days**, how many standard drinks did you have each day?<br>*Don't Know 77*	Monday	A11a	
	Tuesday	A11b	
	Wednesday	A11c	
	Thursday	A11d	
	Friday	A11e	
	Saturday	A11f	
	Sunday	A11g	
I have just asked you about your consumption of alcohol during the past 7 days. The questions were about alcohol in general, while the next questions refer to your consumption of homebrewed alcohol, alcohol brought over the border/from another country, any alcohol not intended for drinking or other untaxed alcohol. Please only think about these types of alcohol when answering the next questions.			
**Question**	**Response**	**Code**	**Skip**

During the **past 7 days**, did you consume any **homebrewed** alcohol; any alcohol **brought over the border/from another country**, any alcohol **not intended for drinking** or other **untaxed** alcohol?(like Tella, Teji, Yehabesha Areki, …etc)	Yes 1<br>No 2	A12	If no, ➔ A14
On average, **how many standard drinks** of the following did you consume **during the past 7 days**? Don't know 77	Homebrewed spirits, e.g. moonshine	A13a	
	Homebrewed beer or wine, e.g. Tella, Teji or Araki	A13b	
	Alcohol brought over the border/from another country	A13c	
	Alcohol not intended for drinking, e.g. alcohol-based medicines, perfumes, after shaves	A13d	
	Other untaxed alcohol in the country	A13e	
**EXPANDED: Alcohol Consumption**			
During the **past 12 months,** how often have you found that you were not able to stop drinking once you had started?	Daily or almost daily 1	A14	
	Weekly 2		
	Monthly 3		
	Less than monthly 4		
	Never 5		
During the **past 12 months,** how often have you failed to do what was normally expected from you because of drinking?	Daily or almost daily 1	A15	
	Weekly 2		
	Monthly 3		
	Less than monthly 4		
	Never 5		
During the **past 12 months,** how often have you	Daily or almost daily 1	A16	

needed a first drink in the morning to get yourself going after a heavy drinking session?	Weekly 2<br>Monthly 3<br>Less than monthly 4<br>Never 5		
**CORE: Diet**			
The next questions ask about the fruits and vegetables that you usually eat. I have a nutrition card here that shows you some examples of local fruits and vegetables. Each picture represents the size of a serving. As you answer these questions, please think of a typical week in the last year.			
**Question**	**Response**	**Code**	**Skip to**
In a typical week, on how many days do you **eat fruit**?	Number of days…………… Don't Know ----77	D1	if Zero days, ➔ D3
How many **servings** of fruit do you eat on **one** of those days?(serving in this case refers to slice or one full fruit e.g. orange, banana, mango, ...)	Number of servings----------<br>Don't Know--------77	D2	
In a typical week, on how many days do you **eat vegetables**?	Number of days ---------------<br>Don't Know ------77	D3	
How many **servings** of vegetables do you eat on one of those days? (serving in this case refers to cups or "**Chilfa"**) of vegetable stews)	Number of days -----------<br>Don't Know -----77	D4	
**Dietary salt**			
With the next questions, we would like to learn more about salt in your diet. Dietary salt includes ordinary table salt, unrefined salt such as sea salt, iodized salt, salty stock cubes and powders, and salty sauces such as soya sauce, fish sauce, tomato sauce, or Magi Mereq. The following questions are on adding salt to the food right before you eat it, on how food is prepared in your home, on eating processed foods that are high in salt, and questions on controlling your salt intake. Please answer the questions, even if you consider yourself to eat a diet low in salt.			
How often do you **add salt or a salty sauce, such as**	Always 1	D7	

**soya sauce** to your food right before you eat it or as you are eating it? (Select only one)	Often 2<br>Sometimes 3<br>Rarely 4<br>Never 5<br>Don't know 77		
How often is **salt, salty seasoning or a salty sauce added** in cooking or preparing foods in your household?	1 Always<br>2 Often<br>3 Sometimes<br>4 Rarely<br>5 Never<br>Don't know---77	D8	
How often do you eat **processed food high in salt**? By processed food high in salt, I mean foods that have been altered from their natural state, such as packaged salty snacks, canned salty food, including pickles and preserves, salty food prepared at a fast food restaurant, cheese, bacon and processed meat *[chips]*.	1 Always<br>2 Often<br>3 Sometimes<br>4 Rarely<br>5 Never<br>Don't know---77	D9	
**How much salt or salty sauce** do you think you consume?	1 Far too much<br>2 Too much<br>3 Just the right amount<br>4 Too little<br>5 Far too little<br>Don't know---77	D10	
**The next questions ask about the oil or fat that is most often used for meal preparation in your household, and about meals that you eat outside a home.**			

What type of **oil or fat is most often** used for meal preparation in your household?	1) Liquid vegetable oil<br>2) Solid vegetable oil(e.g. Viking, Chief...)<br>3) Sesame/Nug oil/Sun flower<br>4) Sheno Lega, Shadi Lega<br>5) Natural butter<br>6) Margarine<br>7) Other<br>8) Other, specify-----------<br>9) None in particular<br>10) None used<br>77)Don't know	D15	if other, ➔<br>D15 other
	Other ----------	D15 other	
On average, how many meals per week do you eat that were not prepared at a home? By meal, I mean breakfast, lunch and dinner.	Number<br>Don't know 77	D16	
**CORE: Physical Activity**			
Next I am going to ask you about the time you spend doing different types of physical activity in a typical week. Please answer these questions, even if you do not consider yourself to be a physically active person. Think first about the time you spend doing work. Think of work as the things that you have to do such as paid or unpaid work, study/training, household chores, harvesting food/crops, fishing or hunting for food, seeking employment. In answering the following questions 'vigorous-intensity activities' are activities that require hard physical effort and cause large increases in breathing or heart rate, 'moderate-intensity activities' are activities that require moderate physical effort and cause small increases in breathing or heart rate.			
**Question**	**Response**	**Code**	**Skip to**
**Work**			

QUESTIONNAIR ID /

Does your work involve vigorous-intensity activity that causes large increases in breathing or heart rate like [carrying or lifting heavy loads, digging or construction work] for at least 10 minutes continuously?	Yes 1<br>No 2	P1	if no, ➔ P4
In a typical week, on how many days do you do vigorous-intensity activities as part of your work?	Number of days -------	P2	
How much time do you spend doing vigorous-intensity activities at work on a typical day?	Hours:-- minutes---	P3 (a-b)	
Does your work involve moderate-intensity activity, that causes small increases in breathing or heart rate such as brisk walking [or carrying light loads] for at least 10 minutes continuously?	Yes 1<br>No 2	P4	if no, ➔ P7
In a typical week, on how many days do you do moderate-intensity activities as part of your work?	Number of days	P5	
How much time do you spend doing moderate-intensity activities at work on a typical day?	Hours:-- minutes---	P6 (a-b)	
**Travel to and from places**			
The next questions exclude the physical activities at work that you have already mentioned. Now I would like to ask you about the usual way you travel to and from places. For example to work, for shopping, to market, to place of worship. *[to church]*			
Do you walk or use a bicycle *(pedal cycle)* for at least 10 minutes continuously to get to and from places?	Yes 1<br>No 2	P7	if no, ➔ P10
In a typical week, on how many days do you walk or bicycle for at least 10 minutes continuously to get to and from places?	Number of days per week------	P8	
How much time do you spend walking or bicycling for travel on a typical day?	Hours:------- minutes---	P9 (a-b)	
**Recreational activities**			
The next questions exclude the work and transport activities that you have already mentioned. Now I would like to ask you about			

sports, fitness and recreational activities (leisure), *[Jim]*.			
Do you do any vigorous-intensity sports, fitness or recreational *(leisure)* activities that cause large increases in breathing or heart rate like *[running or football, aerobics, fast bicycling, fast simming]* for at least 10 minutes continuously? *[Jim, tennis…]*	Yes 1<br>No 2	P12	if no, ➔<br>P15
In a typical week, on how many days do you do vigorous-intensity sports, fitness or recreational *(leisure)* activities?	Number of days	P13	
How much time do you spend doing vigorous-intensity sports, fitness or recreational activities on a typical day?	Hours:---- minutes---	P14(a-b)	
Do you do any moderate-intensity sports, fitness or recreational *(leisure)* activities that cause a small increase in breathing or heart rate such as brisk walking, *[cycling, swimming, and volleyball]* for at least 10 minutes continuously?	Yes 1<br>No 2	P15	if no, ➔<br>P18
In a typical week, on how many days do you do moderate-intensity sports, fitness or recreational *(leisure)* activities?	Number of days	P16	
How much time do you spend doing moderate-intensity sports, fitness or recreational *(leisure)* activities in a typical day?	Hours:-- minutes---	P17<br>(a-b)	
**EXPANDED: Physical Activity**			
**Sedentary behavior/ Time spent sitting**			
The following question is about sitting or reclining at work, at home, getting to and from places, or with friends, including time spent sitting at a desk, sitting with friends, traveling in a car, bus, train, reading, playing cards or watching television, but do not include time spent sleeping.			

How much time do you usually spend sitting or reclining on a typical day?	Hours:-- minutes---	P18 (a-b)	
**Other life style questions**			
**CORE: History of Raised Blood Pressure**			
Have you ever had your blood pressure measured by a doctor or other health worker?	Yes 1<br>No 2	H15	if no, ➔ H24
Have you ever been told by a doctor or other health worker that you have raised blood pressure or hypertension?	Yes 1<br>No 2	H16	if no, ➔ H24
Have you been told in the past 12 months?	Yes 1<br>No 2	H18	
In the past two weeks, have you taken any drugs (medication) for raised blood pressure prescribed by a doctor or other health worker?	Yes 1<br>No 2	H19	
Have you ever seen a traditional healer for raised blood pressure or hypertension?	Yes 1<br>No 2	H22	
Are you currently taking any herbal or traditional remedy for your raised blood pressure?	Yes 1<br>No 2	H23	
Is anyone in your family hypertensive?	Mother----------------------3<br>Brothers and sisters-------4<br>Children --------------------5<br>No one----------------------6<br>Don't know-----------------77	H24	
**CORE: History of Diabetes**			
**Question**	**Response**	**Code**	**Skip**
Have you ever had your blood sugar measured by a doctor or other health worker?	Yes 1<br>No 2<br>Don't know-----88	H36	if no, ➔ H43

Have you ever been told by a doctor or other health worker that you have raised blood sugar or diabetes?	Yes 1<br>No 2	H37a	if no, ➔ H43
Have you been told in the past 12 months?	Yes 1<br>No 2	H37b	
In the past two weeks, have you taken any drugs (medication) for diabetes prescribed by a doctor or other health worker?	Yes 1<br>No 2	H38	
Are you currently taking insulin for diabetes prescribed by a doctor or other health worker?	Yes 1<br>No 2	H39	
	Other specify--------	H40 other	
Have you ever seen a traditional healer for diabetes or raised blood sugar?	Yes 1<br>No 2	H41	
Are you currently taking any herbal or traditional remedy for your diabetes?	Yes 1<br>No 2	H42	
Is anyone in your family diabetes?	Parents----------------------1<br>Father-----------------------2<br>Mother----------------------3<br>Brothers and sisters-------4<br>Children --------------------5<br>No one----------------------6<br>Don't know----------------77	H43	
**CORE: History of Raised Total Cholesterol**			
**Question**	**Response**	**Code**	**Skip**
Have you ever had your cholesterol (fat levels in your blood) measured by a doctor or other health worker?	Yes 1<br>No 2<br>Don't know-----88	H46	if no, ➔ H51
Have you ever been told by a doctor or other health worker that you have raised cholesterol?	Yes 1<br>No 2	H47a	if no, ➔ H51
Have you been told in the past 12 months?	Yes 1	H47b	

	No 2		
In the past two weeks, have you taken any oral treatment (medication) for raised total cholesterol prescribed by a doctor or other health worker?	Yes 1<br>No 2	H48	
Have you ever seen a traditional healer for raised cholesterol?	Yes 1<br>No 2	H49	
Are you currently taking any herbal or traditional remedy for your raised cholesterol?	Yes 1<br>No 2	H50	
Is anyone in your family dyslipidemic?	Parents---------------------1<br>Father----------------------2<br>Mother---------------------3<br>Brothers and sisters-------4<br>Children --------------------5<br>No one---------------------6<br>Don't know----------------77	H51	
**Step 2 Physical Measurements**			
**CORE: Blood Pressure**			
**Question**	**Response**	**Code**	**Skip**
Interviewer ID	-----------	M1	
Device ID for blood pressure	-----------	M2	
Cuff size used	1) Small<br>2) Medium<br>3) Large	M3	
Reading 1	Systolic (mmHg)----------	M4a	
	Diastolic (mmHg)----------	M4b	
Reading 2	Systolic (mmHg)------------	M5a	
	Diastolic (mmHg)-----------	M5b	
Reading 3	Systolic (mmHg)-------------	M6a	
	Diastolic (mmHg)-----------	M6b	

During the past two weeks, have you been treated for raised blood pressure with drugs (medication) prescribed by a doctor or other health worker?	Yes 1<br>No 2	M7	
**CORE: Height and Weight**			
Interviewer ID	---------	M8	
Device IDs for height and weight	Height -------<br>Weight ------	M9a<br>M9b	
Height	in Centimeters (cm-------	M10	
Weight<br>*If too large for scale 666.6*	in Kilograms (kg)-------	M11	
**CORE: Waist**			
Device ID for waist	-----------	M12	
Waist circumference	in Centimetres (cm)	M13	
**EXPANDED: Hip Circumference and Heart Rate**			
Hip circumference	in Centimeters (cm)	M14	
Heart Rate			
Reading 1	Beats per minute---------------	M15a	
Reading 2	Beats per minute---------------	M15b	
Reading 3	Beats per minute----------------	M15c	
**Step 3 Biochemical Measurements**			
**CORE: Blood Glucose**			
**Question**	**Response**	**Code**	**Skip**
During the past 12 hours have you had anything to eat or drink, other than water?	Yes 1<br>No 2	B1	
Technician ID	--------	B2	
Device ID	--------	B3	
Time of day blood specimen taken (24 hour clock)	Hours-----, mins-----	B4	
Fasting blood glucose	*mmol/l*------------	B5	

[CHOOSE ACCORDINGLY: mmol/l OR mg/dl]	*mg/dl*--------------		
Today, have you taken insulin or other drugs (medication) that have been prescribed by a doctor or other health worker for raised blood glucose?	Yes 1<br>No 2	B6	
**CORE: Blood Lipids**			
Device ID	----------	B7	
Participant ID number		B8	
Sub-city		B9	
Sample ID		B10	
Total cholesterol<br>*[CHOOSE ACCORDINGLY: mmol/l OR mg/dl]*	*mmol/l*------------<br>*mg/dl*--------------	B11	
During the past two weeks, have you been treated for raised cholesterol with drugs (medication) prescribed by a doctor or other health worker?	Yes 1<br>No 2	B12	
**EXPANDED: Triglycerides and HDL Cholesterol**			
**Question**	**Response**	**Code**	**Skip**
Triglycerides<br>*[CHOOSE ACCORDINGLY: mmol/l OR mg/dl]*	*mmol/l*------------<br>*mg/dl*--------------	B13	
HDL Cholesterol<br>*[CHOOSE ACCORDINGLY: mmol/l OR mg/dl]*	*mmol/l*------------<br>*mg/dl*--------------	B14	
