# Supplementary material for: Prevalence and risk factors of hypertension among adults: A community based study in Addis Ababa, Ethiopia
Source: PLoS One. 2021 Apr 1;16(4):e0248934. doi: 10.1371/journal.pone.0248934 (PMC8016337; doi:10.1371/journal.pone.0248934)
Supplement: S2 Annex — (PDF) [file pone.0248934.s003.pdf]

የመጠይቅ መለያ ኮድ | | | | |

Annex II ፡- Amharic version questionnaire

የስምምነት ቅጽ

አዲስ አበባ ዩኒቨርሲቲ

ጤና ሳይንስ ኮሌጅ

የኅብረተሰብ ጤና ትምህርት ቤት

ይህ ቃለ መጠይቅ በአዲስ አበባ ከተማ የሚኖሩ አዋቂዎች ላይ ተላላፊ ያልሆኑ በሽታዎችን ማለትም የደም ግፊት፣ የስ≤ር ህመም እንዲሁም በደም ውስጥ የቅባት መጠን አለመመጣጠንን ስርጭት እና መንስኤ ሊሆኑ የሚችሉ ምክንያቶችን ለማጥናት የተዘጋጀ ነዉ፡፡

የመጠይቅ መለያ ኮድ | | | | | |

የዳሰሳ ጥናቱ የሚፈልገው መረጃ፡-

**መረጃው የሚሰበሰብበት ቦታና ቀን**	**ምላሽ/ መልስ**	**ኮድ**	**ማለፍ/ መዝለል**
የአካባቢው/ ስፍራው መለያ ቁጥር	---------------	I1	
የክፍለ ከተማ ስም	---------------	I2	
የሰፈር/ የመንደር ስም	---------------	I3	
የጠያቂው ሙሉ ስም እና መለያ ቁጥር	---------------	I4	
መጠይቁ ሙሉውኑ ተሞልቶ ያለቀበት ቀን	ቀን-----------ወር-------------ዓ.ም--- -----------	I5	
**ፍቃደኝነት፣ የተጠየቁበት ቋንቋና ስም**			
ይሁንታን ስለመጠየቅ እና ስለጥናቱ ማብራሪያ ስለመስጠቱ	1. ተስማምተዋል 2.አልስማሙም ; ካልተስማሙ ማቋረጥ	I6	
የተጠየቁበት ቋንቋ	አማርኛ	I7	
የተጠየቁበት ሰአት	ሰአት------------ደቂቃ-----------	I8	
የተሳታፊ የሚስጥር ቁጥር	----------------------	I9	
የተሳታፊው ሙሉ ስም	--------------------------	I10	
የተሳታፊው አድራሻ ወይም ስልክ ቁጥር	-------------------------	I11	

ክፍል 1 ግለሰባዊ መረጃዎች

ቁልፍ ግለሰባዊ መረጃዎች

**ተ/ቁ**	**ጥያቄ**	**ምላሽ/ መልስ**	**ኮድ**	**ማለፍ/ መዝለል**
1	ፆታ	ወንድ----------------1<br>ሴት------------------2	C1	
2	የልደት ቀንዎ መቼ ነው?	ቀን--------ወር-------------ዓ.ም---------	C2	ካወቁት → ተ/ቁ 4
3	እድሜዎ ስንት ነው?	---------------አመት	C3	
4	የትውልድ ቦታዎ የት ነው?	ገጠር----------------------------1<br>ከተማ----------------------------2<br>አላውቀውም---------------------3	C4	
5	አዲስ አበባ ከተማ ውስጥ ለስንት አመት ኖረዋል?	---------------ወራት<br>---------------አመታት<br>አዲስ አበባ ነው የተወለድኩት-------1	C5	

6	በአጠቃላይ መደበኛ ትምህርት የተማሩት ለስንት አመታት ነው? መዋዕለ ህፃናትን ወይም ቄስ ትምህርት ቤትን ሳይጨምር	---------------------------አመት<br>አላስታውስም--------88	C6	
የተብራራ መጠይቅ፤ ግልሰባዊ መረጃዎች				
8	መደበኛ ትምህርት ተከታትለዋል?	አዎ -------------1<br>የለም------------2	C8	የለም → ተ/ቁ 10
9	ከፍተኛ የትምህርት ደረጃዎ ስንት ነው?	የመጀመሪያ ደረጃ (1-8)----------------1<br>ሁለተኛ ደረጃ (9-10)------------------2<br>መሰናዶ (11-12)-------------------------3<br>የሙያ ትምህርት------------------------4<br>ኮሌጅ እና ከዚያ በላይ--------------5<br>መልስ የለኝም-------------------------88	C9	
10	የየትኛው ብሄር አባል ነዎ?	አማራ----------------------------------1<br>ኦሮሞ----------------------------------2<br>ጉራጌ----------------------------------3<br>ትግራይ--------------------------------4<br>ሌላ ----------------------------------5<br>ሌላ ካለ ይጥቀሱ-------------<br>መመለስ አልፈልግም----------------88	C10	
11	የትዳር ሁኔታዎ እንዴት ነው?	ምንም ያላገቡ--------------------------1<br>ያገቡ-----------------------------------2<br>የተለያዩ--------------------------------3<br>የተፋቱ--------------------------------4<br>የትዳር አጋራቸውን በሞት ያጡ-------5<br>መልስ የለኝም-----------------------88	C11	
12	ሃይማኖትዎ ምንድነው?	ኦርቶዶክስ-----------------------------1<br>ሙስሊም------------------------------2<br>ፕሮቴስታንት--------------------------3<br>ካቶሊክ-------------------------------4<br>ሌላ ----------------------------------5<br>ሌላ ካለ ይጥቀሱ-------------	C12	
13	ከሚከተሉት የማብራሪያ ሐረጎች ባለፉት 12 ወራት የትኛው መልስ የነበሩበትን የስራ ሁኔታ በሚገባ ይገልፃል?	የመንግስት ሰራተኛ----------------------1<br>መንግስታዊ ያልሆኑ ድርጅቶች ተቀጣሪ--2<br>በግል ተዳዳሪ---------------------------3<br>ተማሪ-----------------------------------4<br>የቤት እመቤት--------------------------5<br>የቀን ሰራተኛ---------------------------6<br>ነጋዴ------------------------------------7<br>ስራ የሌለው ግን መስራት የሚችል-----8<br>ስራ የሌለውና መስራት የማይችል------9<br>ጡረተኛ---------------------------------10<br>ሌላ -------------------------------------11<br>ሌላ ካለ ይጥቀሱ-------------	C13	

14	በሚኖሩበት ቤት ውስጥ ከእርሰዎ ጋራ በተጨማሪ ዕድሜአቸው ከ 18 ዓመት በላይ የሆናቸው የቤተሰብ አባላት ቁጥር ምን ያህል ነው?	የሰዎች ብዛት በቁጥር--------------	C14	
15	ከእርሰዎ ጋራ የቤተሰብዎ ብዛት ስንት ነው?	የሰዎች ብዛት በቁጥር--------------	C15	
16	ወርሃዊ የመተዳደሪያ ገቢዎን ሊገልፁልን ይዎዳሉ?	----------------------------ብር<br>ምንም ገቢ የለኝም -------98<br>ለመመለስ አልፈልግም----88	C16	
**ክፍል 2- የባህሪ መለያዎች**				
**ቁልፍ - ትንባሆ/ ሲጃራ ማጨስን በተመለከተ**				
**ከዚህ ቀጥሎ ሲጃራ ወይም ትንባሆ ማጨስን በተመለከተ እጠይቀዎታለሁ**				
**ተ/ቁ**	**ጥያቄ**	**ምላሽ/ መልስ**	**ኮድ**	**ማለፍ/ መዝለል**
17	ባሁኑ ወቅት ሲጃራ ያጨሳሉ? ለምሳሌ፡- ሲጃራ፣ ፒፓ፣ ጋያ	አጨሳለሁ-----------------1<br>አላጨስም----------------2	T1	አላጨስም → ተ/ቁ 24
18	ባሁኑ ወቅት ሲጃራ በየቀኑ ያጨሳሉ?	አጨሳለሁ-----------------1<br>አላጨስም----------------2	T2	
19	ሲጃራ ማጨስ የጀመሩት በስንት አመትዎ ነበር?	---------------------አመቴ<br>እድሜዬን አላስትውስም------------88	T3	
20	ለምን ያህል ዘመን እንዳጨሱ ያስታውሳሉ?	-------------አመት<br>------------ወራት<br>------------ሳምንታት<br>አላስትውስም------------88	T4a<br>T4b<br>T4c	

ተ/ቁ	ጥያቄ	ምላሽ/ መልስ	**በየቀኑ**	**በየሳምንቱ**	ኮድ	ማለፍ/ መዝለል
21	በአማካይ በእያንዳንዱ ቀን ወይም ሳምንት ጊዜ ውስጥ የሚከተሉትን ምርቶች ምን ያህል ያጨሳሉ ወይም ይጠቀማሉ?					
		በፋብሪካ የተመረቱ ሲጃራዎችን			T5a/Tsaw	
		በእጅ የሚጠቀለሉ ሲጃራዎችን			T5b/Tsbw	
		ፒፓዎችን			T5c/Tscw	
		በትንባሆ ቅጠል የተጠቀለሉ ሲጃራዎችን			T5d/Tsdw	
		ሺሻዎችን በቁጥር			T5e/Tsew	
		ሌላ			T5f/Tsfw	
		ሌላ ካለ ይግለጹ				
		አላውቀውም--88				

የመጠይቅ መለያ ኮድ | | | | | |

22	ባለፉት 12 ወራት ማጨስ ለማቆም ሙከራ አድርገዋል?	አዎ -------------------1<br>የለም አላደረኩም-----2	T6	
23	ባለፉት 12 ወራት ወደ ህክምና ሲሄዱ የጤና ሙያተኞች ማጨስ እንዲያቆሙ ነግረዎወት ነበር?	አዎ--------------------1<br>የለም-----------------2<br>በ12 ወራት ውስጥ ወደ ሃኪም ቤት አልሄድኩም-------3	T7	
24	በህይወት ዘመንዎ የትምባሆ ምርቶችን ተጠቅመው ያውቃሉ?	አዎ---------------------1<br>የለም ------------------2	T8	የለም →<br>ተ/ቁ 28
25	በህይወት ዘመንዎ የትምባሆ ምርቶችን በየቀኑ ይጠቀሙ ነበር?	አዎ----------------------1<br>የለም--------------------2	T9	

የተብራራ መጠይቅ ፤ ማጨስዎን በተመለከተ

**ተ/ቁ**	**ጥያቄ**	**ምላሽ/መልስ**	**በየቀኑ**	**በየሳምንቱ**	**ኮድ**	**ማለፍ/ መዝለል**
26	ማጨስ ሲያቆሙ እድሜዎ ስንት ነበር?	-------------------እድሜ<br>አላውቀውም--------88			T10	
27	ማጨስ ካቆሙ ምን ያህል ጊዜ ሆነዎት?	----------------አመት			T11a	
		---------------ወራት			T11b	
		---------------ሳምንታት			T11c	
28	በአሁኑ ወቅት ጭስ የሌላቸውን የትምባሆ ምርቶች ይጠቀማሉ? ለምሳሌ፤ የሚሸተት፣ የሚታኘክ፣ በምግብ የሚወሰድ	አዎ------------------------------1<br>የለም----------------------------2			T12	የለም →<br>ተ/ቁ 31
29	በአሁኑ ወቅት ጭስ የሌላቸውን የትምባሆ ምርቶች በየቀኑ ይጠቀማሉ? ለምሳሌ፤ የሚሸተት፣ የሚታኘክ፣ በምግብ የሚወሰድ	አዎ------------------------------1<br>የለም----------------------------2			T13	
30	የሚጠቀሙ ከሆነ በአማካይ በቀን ምን ያህል ጊዜ ይጠቀማሉ?<br>(ከቀን ያነሰ ከሆነ በሳምንት ይያዝ)		**በየቀኑ**	**በየሳምንቱ**		
		በአፍ የሚማግ			T14a/T14aw	
		በአፍንጫ የሚሸተት			T14b/T14bw	
		የሚታኘክ			T14c/T14cw	
		ሌላ			T14d/T14dw	
		ሌላ ካለ ይጥቀሱ--------------				
		ካላዎቁት 88 ይፃፍ				
31	በህይወት ዘመንዎ ጭስ የሌላቸው የትምባሆ ምርቶችን ተጠቅመው ያውቁ ነበር? ለምሳሌ፤ የሚሸተት፣ የሚታኘክ፣ በምግብ የሚወሰድ	አዎ-----------------------------------1<br>የለም--------------------------------2			T15	የለም →<br>ተ/ቁ 33

32	በህይወት ዘመንዎ ጭስ የሌላቸው የትምባሆ ምርቶች በየቀኑ ተጠቅመው ያውቁ ነበር? ለምሳሌ፤ የሚሸተት፣ የሚታኘክ፣ በምግብ የሚወሰድ	አዎ-----------------------------------1<br>የለም---------------------------------2	T 16	
33	በለፉት 30 ቀናት በቤትዎ ውስጥ ሲጃራ የሚያጨስ ሰው ነበር? (ሲጃራ የማያጨሱ ከሆነ)	አዎ-----------------------------1<br>የለም---------------------------2<br>አላስታውስም-----------------88	T 17	
34	በለፉት 30 ቀናት እርስዎ በሚገኙበት ጠባብ ክፍል ውስጥ ሲጃራ የሚያጨስ ሰው ነበር? ለምሳሌ፣ በሚኖሩበት ህንፃ፣ በሚሰሩበት ስፍራ፣ በቢሮዎ ውስጥ	አዎ---------------------------------1<br>የለም-------------------------------2<br>በተጣበበ ቦታ ስራ አልሰራም-----3	T18	
35	በአሁኑ ወቅት ጫት ይቅማሉ?	አዎ-----------------------------1<br>የለም--------------------------2<br>መመለስ አልፈልግም---------88	T19	የለም ⟶ ተ/ቁ 42
36	በአማካይ በአንድ ወር ጊዜ ውስጥ ለምን ያህል ቀናት ጫት ይቅማሉ?	በየቀኑ/በየቀኑ በሚያስብል ሁኔታ-----1<br>በየሳምንቱ----------------------------2<br>በየወሩ--------------------------------3<br>ሌላ ---------------------------------4<br>ሌላ ካለ ይጥቀሱ--------------	T20	
37	በአንድ ጊዜ በሚቅሙበት ወቅት ምን ያህል ዙርባ ወይም እስር ይቅማሉ?	------------ዙርባ/ እስር	T20a	
38	ጫት መቃም የጀመሩት በስንት አመትዎ ነበር?	------------------------አመቴ	T21	
39	ለምን ያህል ዘመን/ጊዜ ጫት ቅመዋል?	--------------------------ሳምንታት<br>-----------------------------ወራት<br>----------------------------አመታት	T22	
40	በህይወት ዘመንዎ ጫት ቅመው ያውቃሉ?	አዎ----------------------1<br>የለም-------------------2<br>መልስ የለኝም---------88	T23	የለም ⟶ ተ/ቁ 43
41	ጫት መቃም ያቆሙት በስንት አመትዎ ነበር?	-------------------አመቴ	T24	
42	ጫት መቃም ካቆሙ ምን ያህል ዘመን/ ጊዜ ሆኖዎታል?	------------------ሳምንታት<br>------------------ወራት<br>------------------አመታት	T25	

**ቁልፍ፣ የአልኮል አወሳሰድን በተመለከተ**				
**የሚከተሉት መጠይቆች የአልኮል ፍጆታወን እና አወሳሰድዎን የሚመለከቱ ይሆናሉ**				
**ተ/ቁ**	**ጥያቄ**	**ምላሽ/መልስ**	**ኮድ**	**ማለፍ/ መዝለል**
43	የአልኮል መጠጦችን ይጠጣሉ? ለምሳሌ፡- ቢራ፣ ወይን፣ አረቂ፣ውስኪ ወዘተ	አዎ------------------------1<br>የለም---------------------2	A1	የለም ⟶ ተ/ቁ 61

44	በለፉት 12 ወራት የአልኮል መጠጦችን ወስደው/ ጠጥተው ያውቃሉ?	አዎ---------------------1<br>የለም-------------------2	A2	አዎ<br>→<br>ተ/ቁ 46
45	መጠጥ መጠጣትን በጤናዎ ምክንያት አቁመዋል? ምን ማለቱ ነው በጤናዎ ላይ አሉታዊ ተፅእኖ በማሳደሩ? (ወይም የጤና ሙያተኛ በሰጠዎ ምክር የተነሳ)	አዎ----------------------1<br>የለም-------------------2	A3	አዎ<br>→<br>ተ/ቁ 61
46	በለፉት 12 ወራት በተደጋጋሚ በሚያስብል ስሌት ቢያንስ አንድ መደበኛ/ የአልኮል መጠን ያለው መጠጥን ለምን ያህል ጊዜ አዘውትረው ነበር?	በየቀኑ----------------------------1<br>በሳምንት ለ5 ወይም 6 ቀናት----2<br>በሳምንት ከ3 እስከ 4 ቀናት------3<br>በሳምንት 1 ወይም 2 ቀናት-------4<br>በወር ከ1 እስከ 3ቀናት------------5<br>በወር ከ 1 ቀን ያነሰ ቀናት---------6	A4	
47	በለፉት 30 ቀናት የአልኮል መጠጥ ወስደው ወይም ጠጥተው ያውቃሉ?	አዎ----------------------------1<br>የለም--------------------------2	A5	የለም<br>→<br>ተ/ቁ 60
48	በለፉት 30 ቀናት በምን ያህል የተለያዩ ዝግጅቶች ላይ ቢያንስ አንድ መደበኛ የአልኮል መጠጥ ጠጥተዋል?	በቁጥር ይግለፁ-----------------<br>አላውቅም--------------------88	A6	
49	በለፉት 30 ቀናት በአማካይ ለምን ያህል ጊዜ መደበኛ የአልኮል ይዘት ያላቸውን መጠጦች በግብዣ ኩነቶች ጠጥተዋል?	በቁጥር ይግለፁ-----------------<br>አላውቅም-------------------88	A7	
50	በለፉት 30 ቀናት ብዛት ያለው መደበኛ የአልኮል መጠጥ በአንድ አጋጣሚ ጠጥተው ከሆነ የሁሉንም መጠጥ አይነት የጠጡትን ደምረው ቢነግሩኝ?	ትልቁ የጠጡት የመጠጥ ቁጥር------<br>አላውቅም---------------------88	A8	
51	በለፉት 30 ቀናት ስድስት እና ከዚያ በላይ የሆኑ መደበኛ የአልኮል መጠጦችን በአንድ ግብዣ ብቻ ለምን ያህል ጊዜ ተጠቅመዋል?	የተጠቀሙበት የጊዜ መጠን በቁጥር----<br>አላውቅም-----------------88	A9	
52	ለወንዶች፤ አምስት እና ከአምስት በላይ	የተጠቀሙበት የጊዜ መጠን በቁጥር----<br>አላውቅም------------------88	A10	
53	ለሴቶች፤ አራት እና ከአራት በላይ	የተጠቀሙበት የጊዜ መጠን በቁጥር----<br>አላውቅም-----------------------88	A11	
54	ባለፉት ሰባት ቀናት ውስጥ ምን ያህል መደበኛ የአልኮል መጠጦችን በየእለቱ ጠጥተዋል?	ሰኞ	A13a	
		ማክሰኞ	A13b	
		ረበዕ	A13c	
		ሐሙስ	A13d	
		አርብ	A13e	
		ቅዳሜ	A13f	
		እሁድ	A13g	
		አላውቅም--------------------88		
እስካሁን ድረስ ላለፉት ሰባት ቀናት ስለጠጡት አልኮሆል ነበር ስንነጋገር የነበረው በቀጣይ ልጠይቀዎት የምፈልገው ሁሉንም አይነት አልኮሎች ሳይሆን በቤት ውስጥ የሚዘጋጁ፣ ከውጭ አገር የሚገቡ የአልኮል መጠጦችን፣ በተለይም የመንግስት ቀረጥ ሳይከፈልባቸው ለመጠጥ ፍጆታ የማይውሉ ደንበር አቋርጠው የሚገቡ መጠጦችን በተመለከተ ይሆናል፡፡ ስለሆነም ጥቂት ጊዜ ወስደው ያስቡበት እና				

የመጠይቅ መለያ ኮድ

ጥያቄዎችን ይመልሱልኛል፡፡				
**ተ/ቁ**	**ጥያቄ**	**ምላሽ/መልስ**	**ኮድ**	**ማለፍ/ መዝለል**
55	ላለፉት ሰባት ቀናት በቤት ውስጥ የተዘጋጀ፣ ለመጠጥ ፍጆታ ፍቃድ ሳይኖራቸው ቀረጥ ሳይከፈልባቸው ደንበር አቋርጠው የገቡ መጠጦችን ጠጥተዋል? ለምሳሌ ጠላ፣ ጠጅ፣ የሃበሻ አረቂ	አዎ-----------------1<br>የለም--------------2	A14	የለም → ተ/ቁ 57
56	ባለፉት ሰባት ቀናት በአማካይ ምን ያህል መደበኛ የአልኮል መጠጦችን ጠጥተዋል?	በቤት ውስጥ የተዘጋጀ የተጣራ አልኮል ለምሳሌ ጠላ------	A15a	
		በቤት ውስጥ የተዘጋጀ ጠጅ--	A15b	
		በቤት ውስጥ የተዘጋጀ አረቂ----	A15c	
		ከአገር ውጭ የገቡ የአልኮል መጠጦች---------	A15d	
		ለመጠጥ ፍጆታ ያልተፈቀዱ እና ቀረጥ ሳይከፈልባቸው ደንበር አቋርጠው የገቡ መጠጦች------	A15e	
		ሌሎች ታክስ ያለልተከፈለባቸው መጠጦች----------	A15f	
		አላውቅም--------------------88		
**ማበራሪያ የሚጠይቁ ጥያቄዎች፤ የአልኮል መጠጥ አወሳሰደዎን በተመለከተ**				
57	ባለፉት 12 ወራት ከመጠጥ ለመቆጠብ አስበው ሲሞክሩ ለምን ያህል ጊዜ አልቻሉም?	በየቀኑ----------------------1<br>በየሳምንቱ-----------------2<br>በወራት--------------------3<br>ከወር ባነሰ----------------4<br>አልሞከርኩትም---------5	A16	
58	ባለፉት 12 ወራት ለምን ያህል ጊዜ መስራት የፈለጉትን ጉዳይ ለማሳካት አስበው መጠጥ በመጠጣተዎ ምክንያት ብቻ ዳር ያልደረሰልዎ ስራ አለ?	በየቀኑ---------------------1<br>በየሳምንቱ---------------2<br>በወራት-------------------3<br>ከወር ባነሰ--------------4<br>በጭራሽ-----------------5	A17	
59	ባለፉት 12 ወራት መጠጥ ከጠጡ በኋላ የተለመደ ስራዎትን ለመስራት ለምን ያህል ጊዜ አቅቶዎታል?	በየቀኑ--------------------1<br>በየሳምንቱ---------------2<br>በወራት-------------------3<br>ከወር ባነሰ--------------4<br>በጭራሽ-----------------5	A18	

60	ባለፉት 12 ወራት አብዝተው ጠጥተው ካደሩ በኋላ በማለዳ ወደ ስራ ከመግባተዎ በፊት አልኮል መቅመስ ለምን ያህል ጊዜ አስፈልጎዎታል?	በየቀኑ--------------------1<br>በየሳምንቱ---------------2<br>በወራት------------------3<br>ከወር ባነሰ---------------4<br>በጭራሽ------------------5	A19	
ቁልፍ ፣ አመጋገብን በተመለከተ				
የሚቀጥሉት መጠይቆች መሰረት የሚያደርጉት በአመጋገብ ስርአትዎ ውስጥ አትክልትና ፍራፍሬዎችን በዘወትር ገበታዎ ላይ መጠቀምን ይመለከታል፡፡ በካርዱ ላይ ያለው እያንዳንዱ ስዕል ለአገልገሎት ሊውል የሚችለውን የምግቡን መጠን ይገልፃል፡፡ እባክዎ ከባለፈው አመት ትዝታዎ አንድ ሁነኛ ሳምንት አስታውሰው ይንገሩኝ፡፡				
**ተ/ቁ**	**ጥያቄ**	**ምላሽ/ መልስ**	**ኮድ**	**ማለፍ/ መዝለል**
61	በአንድ ሁነኛ ሳምንት ለምን ያህል ቀናት ፍራፍሬ ተመግበዋል?	ቀኖቹን በቁጥር ይጥቀሱ-------<br>አላውቀውም----------------88	D1	ዜሮ ቀን → ተ/ቁ 63
62	ፍራፍሬ በተመገቡበት ባንዱ ቀን ውስጥ ምን ያህል የገበታ አቅርቦትዎ ፍራፍሬዎችን ያካተተ ነበር? (ለምሳሌ፡- የተከፈለ ወይም አንድ ሙሉ ማንጎ፣ ብርቱካን፣ ፓፓዬ የመሳሰሉትን ማለቴ ነው)	የገበታ አቅርቦትዎ በቁጥር------<br>አላውቀውም----------------88	D2	
63	በአንድ ሁነኛ ሳምንት ለምን ያህል ቀናት አትክልቶችን ተመግበዋል?	ቀኖቹን በቁጥር ይጥቀሱ---------<br>አላውቀውም-----------------88	D3	ዜሮ ቀን → ተ/ቁ 65
64	አትክልቶችን በተመገቡበት ባንዱ ቀን ውስጥ ምን ያህል የገበታ አቅርቦትዎ አትክልቶችን ያካተተ ነበር? (ለምሳሌ፡-በጭልፋ፣ በማንኪያ....)	የገበታ አቅርቦትዎ በቁጥር-------<br>አላውቀውም-----------------88	D4	
በምግብ ገበታዎ ውስጥ የጨው አጠቃቀምዎን በተመለከተ				
በሚቀጥሉት መጠይቆች ማተኮር የምፈልገው በምግብዎ ውስጥ ስለሚጠቀሙት የጨው(ጥሬ ጨው) መጠን ነው፡፡ የገበታ ጨው የሚባሉት መደበኛ የገበታ ጨው(የታሸገ)፣ ከውቅያኖስ የሚወጣ ያልተጣራ ጨው፤ አዮዳይዝድ የገበታ ጨው፤ አሞሌ ወይም የደቀቀ አሞሌ፣ የሾርባ ጨው፣ ሶያ የሾርባ ጨው፣ ማጂ መረቅ፣ የቲማቲም ሶስ ወይም የአሳ ሾርባ ጨው ሊሆኑ ይችላሉ፡፡ የሚከተሉት ጥያቄዎች በምግብዎ ውስጥ ስለሚጨምሩት ጨው የሚመለከቱ ናቸው፡፡ ይህም ሲባል ከመመገብዎ በፊት ምግበዎ ውስጥ ስለሚጨምሩት የጨው መጠን፣ ምግብ በቤትዎ እንዴት እንደሚዘጋጅ፣ ከፍተኛ የሆነ የጨው መጠን ያላቸውን የታሸጉ ወይም የተዘጋጁ ምግቦችን እንደሚጠቀሙ፣ በምግበዎ ውስጥ ያለውን የጨው መጠን እንዴት እንደሚቆጣጠሩ የሚሉ ይገኙበታል፡፡ እባክዎ አነስተኛ ጨው ነው በምግቦቹ የምጠቀመው የሚሉ ቢሆን እንኳን ለጥያቄዎች መልስ ይስጡባቸው፡፡				
**ተ/ቁ**	**ጥያቄ**	**ምላሽ/ መልስ**	**ኮድ**	**ማለፍ/ መዝለል**
65	በምግብዎ ውስጥ ጨው፣ የሶያ ጨው፣ የቲማቲም ሶስ ምን ያህል አዘውትረው ይጠቀማሉ ከምግብ በፊት ወይም እየተመገቡ እያለ?(አንድ መልስ ብቻ ይምረጡ)	ሁልጊዜ---------------------1<br>ብዙውን ጊዜ---------------2<br>አንዳንድ ጊዜ------------- 3<br>አልፎ አልፎ-------------- 4<br>በጭራሽ------------------ 5<br>አላውቅም----------------88	D7	

66	በቤትዎ ውስጥ ምግብ በሚሰሩበትና በሚያዘጋጁበት ወቅት ጨዋማ የሆነ፣ የጨው ማጣፈጫ፣ የቲማቲም ሶስ የሆነ ወይም የሾርባ ጨው ምን ያህል ይጨምራሉ?	ሁልጊዜ------------------- 1<br>ብዙውን ጊዜ------------- 2<br>አንዳንድ ጊዜ------------- 3<br>አልፎ አልፎ------------- 4<br>በጭራሽ------------------ 5<br>አላውቅም-----------------88	D8	
67	ከፍተኛ የጨው መጠን ተጨምሮባቸው የተዘጋጁ ምግቦችን ምን ያህል ያዘወትራሉ? ይህን ስል ምግብን ለማቆየት ሲባል ከፍተኛ የጨው መጠን በምግብ ውስጥ ይጨመራል ለምሳሌ፡- የታሸጉ ምግቦች፣ ቱና፣ የድንች ጥብስ፣ ቋንጣ፣ ቺዝ የመሳሰሉትን ለማለት ፈልጌ ነው፡፡	ሁልጊዜ--------------------- 1<br>ብዙውን ጊዜ--------------- 2<br>አንዳንድ ጊዜ--------------- 3<br>አልፎ አልፎ---------------- 4<br>በጭራሽ-------------------- 5<br>አላውቅም-------------------88	D9	
68	የሚጠቀሙት የጨው እና የሶያ ጨው መጠን ምን የህል ነው ብለው ያስባሉ?	እጅግ በጣም ብዙ------------1<br>በጣም ብዙ-------------------2<br>ትክክለኛውን መጠን ---------3<br>አነስተኛ መጠን እጠቀማለሁ--4<br>አልጠቀምም ማለት ይቻላል---5<br>አላውቀውም-----------------88	D10	
**የሚቀጥሉት መጠይቆች በቤተዎ ውስጥ ወይም ከቤትዎ ውጭ ምግብ ሲመገቡ ስላለው የቅባት እና የዘይት አጠቃቀምዎ ላይ ይሆናል**				
**ተ/ቁ**	**ጥያቄ**	**ምላሽ/ መልስ**	**ኮድ**	**ማለፍ/ መዝለል**
69	በአብዛኛው ጊዜ በቤተዎ ውስጥ ምግብ ሲዘጋጅ የሚጠቀሙት ዘይት ወይም ቅባት ምን አይነት እንደሆነ ይምረጡ	የአትክልት ዘይት---------------1<br>ጠጣር(የሚረጋ) የአትክልት ዘይት (ቫይኪንግ፣ ቺፍ)-------2<br>የሰሊጥ/የኑግ/የሱፍ ዘይት-----3<br>ቅቤ---------------------------4<br>ማርጋሪን----------------------5<br>ሽኖ ለጋ/ ሻዲ ለጋ-------------6<br>ሌላ --------------------------7<br>ሌላ ካለ ይጥቀሱ-------------<br>ምንም አልጠቀምም---------98<br>አላውቅም-------------------88	D15	
70	በአማካይ በሳምንት ውስጥ ከቤትዎ ውጭ የተዘጋጁ ምግቦችን ለምን ያህል ጊዜ ይጠቀማሉ? ማለቴ ቁርስ፣ ምሳ ወይም ራት ማለቴ ነው፡፡	በቁጥር ይግለፁ---------------<br>አላውቅውም --------------88<br>አልመገብም-----------------98	D16	
**ቁልፍ፣ የአካል እንቅስቃሴን በተመለከተ**				
ከዚህ ቀጥሎ ደግሞ በሳምንት ውስጥ የተለያዩ ተግባራትን ለመከወን ስለሚያደርጉት አካላዊ እንቅስቃሴ እጠይቀዎታለሁ፡፡ እባክዎ እነዚህን ጥያቄዎች ንቁ የአካል እንቅስቃሴ የማደርግ ሰው አይደለሁም ቢሉ እንኳን ምላሽ ይስጡባቸው፡፡ በመጀመሪያ በስራ ላይ ሆነው ያሳለፉትን ጊዜ ያስታውሱ/ ያስሉ፡፡ያከናወኑት ስራ ክፍያ ያለው ወይም የሌለው ሊሆን ይችላል፤ ማንበብ፣ ትኩረት የሚሻ ምርምር፣ የቤት ውስጥ ስራዎች፣ አዝርእት ማጨድ፣ አሳ ማስገር፣ ስራ ፍለጋ ዞረው ሊሆን ይችላል፡፡ በሚቀጥሉት መጠይቆች ውስጥ "ጥልቅ ትኩረትና አንክሮ የሚጠይቁ ተግባራት" የሚለው ሀረግ ጠንካራ ጉልበት ብሎም ብዙ ትንፋሽ የሚያስወጣ ስራ ወይም ተግባርን ይዎክላል፡፡ "መጠነኛ ትኩረት እና አንክሮ የሚጠይቁ ተግባራት" የሚለው ሀረግ ደግሞ ተግባሩ መለስተኛ ጉልበት ብሎም ብዙ ትንፋሽ የማያስወጣ የሚል እንደሆነ እንዲረዱልኝ እፈልጋለሁ፡፡				
**ተ/ቁ**	**ጥያቄ**	**ምላሽ/ መልስ**	**ኮድ**	**ማለፍ/ መዝለል**

የመጠይቅ መለያ ኮድ | | | | | |

**ስራዎን በተመለከተ**				
71	የእርስዎ ስራ ጠንካራ ጉልበት ብሎም ጥልቅ ትኩረት እና አንክሮ ይጠይቃል? ለማብራራት ያህል ስራዎ ብዙ ትንፋሽ የሚያስወጣና የልብን ምት የሚያስጨምሩ ማለትም(ከባድ ሸክሞችን ማንቀሳቀስ፣ መቆፈር ወይም የግንባታ ስራወችን ያጠቃልላል)? በተጨማሪም ቢያንስ ለ 10 ደቂቃዎች ያለማቋረጥ በስራ ላይ ሊቆዩ ይችላሉ?	አዎ-------------------1<br>የለም-----------------2	P1	የለም ⟶ ተ/ቁ 74
72	ሁነኛ በተባለ አንድ ሳምንት ውስጥ ለምን ያህል ቀናት ጠንካራ ጉልበት፣ ጥልቅ ትኩረትና አንክሮ የሚጠይቅ ስራ ሰርተው ያውቃሉ?	የቀናቱን ቁጥር በአሀዝ ይጥቀሱ--------------	P2	
73	ሁነኛ በተባለ አንድ ቀን ውስጥ ለምን ያህል ሰአት ጠንካራ ጉልበት፣ ጥልቅ ትኩረትና አንክሮ የሚጠይቅ ስራ ይሰራሉ?	ሰአት----------ደቂቃ ------	P3 (a-b)	
74	የእርስዎ ስራ መለስተኛ ጉልበት ብሎም ብዙ ትንፋሽ የማያስወጣ መጠነኛ ትኩረትና አንክሮ የሚፈልግ ስራ ነው? ለምሳሌ ፈጠን ያለ ርምጃ፣ ቀለል ያለ እቃ መሸከም (ቢያንስ ለ 10 ደቂቃ በተከታታይ)	አዎ---------------1<br>የለም ------------2	P4	የለም ⟶ ተ/ቁ 76
75	ሁነኛ በተባለ አንድ ሳምንት ውስጥ ለምን ያህል ቀናት መለስተኛ ጉልበት ብሎም ብዙ ትንፋሽ የማያስወጣ መጠነኛ ትኩረትና አንክሮ የሚፈልግ ስራ ሰርተው ያውቃሉ?	የቀናቱን ቁጥር በአሀዝ ይጥቀሱ--------------	P5	
76	ሁነኛ በተባለ አንድ ቀን ውስጥ ለምን ያህል ሰአት መለስተኛ ጉልበት ብሎም ብዙ ትንፋሽ የማያስወጣ መጠነኛ ትኩረትና አንክሮ የሚፈልግ ስራ ያከናውናሉ?	ሰአት---------ደቂቃ --------------	P6 (a-b)	
**ከቦታ ወደ ቦታ መንቀሳቀስን በተመለከተ**				
አሁን የምጠይቀወት የሚከተሉት ጥያቄዎች ጉልበት የሚጠይቁ ተግባራትን ሳይሆን በተለመደው እለታዊ እንቅስቃሴ ከቦታ ወደ ቦታ ስለሚያደርጉት ጉዞ ነው፡፡ ለምሳሌ ያህል ገበያ ሲገበዩ፣ ወደ ሱፐር ማርኬት ሲሄዱ ወይም ወደ ቤተ እምነት ሲሄዱ ስለሚያደርጓቸው እንቅስቃሴወች እንደማለት ነው፡፡				
**ተ/ቁ**	**ጥያቄ**	**ምላሽ/ መልስ**	**ኮድ**	**ማለፍ/ መዝለል**
77	ከአንድ ቦታ ደርሶ ለመመለስ ቢያንስ 10 ደቂቃወች የሚዎስድ ጉዞን በእግር ወይም ቢስክሌት በመጠቀም ይጓዛሉ?	አዎ--------------------1<br>የለም -----------------2	P7	የለም ⟶ ተ/ቁ 80
78	በአንድ ሁነኛ በተባለ ሳምንት ውስጥ ለምን ያህል ቀናት በእግርዎ ወይም ቢስክሌት በመጠቀም ቢያንስ ለ10 ደቂቃወች የሚዎስድ ጉዞን ተጉዘዋል?	የቀናቱን ቁጥር በአሀዝ ይጥቀሱ--------------	P8	
79	በአንድ ሁነኛ በተባለ እለት ለምን ያህል ሰአት በእግርዎ ወይም ቢስክሌት በመጠቀም ተጉዘዋል?	ሰአት---------ደቂቃ -------------	P9 (a-b)	
**ለመናፈስ ወይም ለመዝናናት የሚደረጉ እንቅስቃሴዎችን በተመለከተ**				
የሚከተሉት ጥያቄዎች ከላይ የተጠቀሱትን የስራ ወይም የጉዞ እንቅሰቃሴወች አይመለከቱም፡፡ ይልቁንም እርስዎ ራስዎን ለማዝናናት መንፈስዎን ለማደስ በሚያደርጓቸው እንቅስቃሴዎች ላይ ትኩረት ያደርጋሉ፡፡ ለምሳሌ ስፓርት፣ የአካል ብቃት እንቅስቃሴ እና ለመናፈስ የሚደረግ ጉዞ የመሳሰሉትን ያጠቃልላል ነገር ግን በቀጣይነት ወይም ያለማቆራረጥ የሚሰሩ ስራዎችን ይመለከታል፡፡(ጂም፣				

ዋና)				
ተ/ቁ	ጥያቄ	ምላሽ/ መልስ	ኮድ	ማለፍ/ መዝለል
80	ለራስዎ በሚሰጡት የመዝናኛ ጊዜ ጉልበት የሚጠይቁ እና ትንፋሽ የሚያበዙ ስፖርቶችን ወይም የአካል ብቃት እንቅስቃሴዎችን ቢያንስ ለተከታታይ አስር ደቂቃ ያደርጋሉ (ለምሳሌ- እግር ኳስ ይጫወታሉ፣ ቴኒስ፣ ሩጫ፣ ፈጣን ዋና፣ ጂም፣በሙዚቃ የታገዘ የሰውነት ማጎልመሻ)	አዎ------------------------1<br>የለም ---------------------2	P12	የለም → ተ/ቁ 83
81	በአንድ ሁነኛ በተባለ ሳምንት ውስጥ ለምን ያህል ቀናት ብርቱ እና አድካሚ ሰፖርት፣ የአካል ብቃት እንቅስቃሴ እና የሚያዝናኑ እንቅስቃሴዎችን ያደርጋሉ?	የቀናቱን ቁጥር በአሀዝ ይጥቀሱ---------------	P13	
82	በአንድ ሁነኛ በተባለ ቀናት ውስጥ ለምን ያህል ሰአት ብርቱ እና አድካሚ ሰፖርት፣ የአካል ብቃት እንቅስቃሴ እና የሚያዝናኑ እንቅስቃሴዎችን ያደርጋሉ?	ሰአት------------------ደቂቃ---------------	P14 (a-b)	
83	መለስተኛ ጉልበት ብሎም ብዙ ትንፋሽ የማያስወጣ መጠነኛ ትኩረትና አንክሮ የሚፈልግ እና ቢያንስ በቀጣይነት ለ10 ደቂቃ የሚሰራ፤ ለምሳሌ ፈጠን ያለ የእግር ጉዞ፣ ሳይክል፣ ዋና፣ የእጅ ኳስ ያካሂዳሉ?	አዎ---------------------------1<br>የለም-------------------------2	P15	የለም → ተ/ቁ 86
84	በአንድ ሁነኛ በተባለ ሳምንት ውስጥ ለምን ያህል ቀናት ብርቱ እና አድካሚ ያልሆኑ ለምሳሌ ሰፖርት፣ የአካል ብቃት እንቅስቃሴ እና የሚያዝናና እንቅስቃሴዎችን ያደርጋሉ?	የቀናቱን ቁጥር በአሀዝ ይጥቀሱ---------------	P16	
85	በአንድ ሁነኛ በተባለ ቀን ውስጥ ለምን ያህል ሰአት ብርቱ እና አድካሚ ያልሆኑ ለምሳሌ ሰፖርት፣ የአካል ብቃት እንቅስቃሴ እና የሚያዝናኑ እንቅስቃሴዎችን ያደርጋሉ?	ሰአት---------------ደቂቃ--------	P17 (a-b)	
**አካላዊ እንቅስቃሴን በተመለከተ የተብራራ ምላሽ የሚጠይቁ ጥያቄዎች**				
**ብዙ እንቅስቃሴ ያለማድረግ ባህሪያት**				
የሚከተሉት ጥያቄዎች ያለምንም እንቅስቃሴ ወይም የጎላ እንቅስቃሴ በማይጠይቁ ለምሳሌ ያህል በቤት ውስጥ መቀመጥ፣ ገደም ማለት፣ በበረንዳ ላይ ዘወርወር ማለት፣ ከጓደኛ ጋራ በጠረጴዛ ዙሪያ ውይይት ማድረግ፣ በመኪና መጓዝ፣ ማንበብ፣ ካርታ መጫወት፣ ቴሌቪዥን መመልከት የመሳሰሉትን እለታዊ ተግባራት ይመለከታል የእንቅልፍ ጊዜን ግን አይጨምርም፡፡				
ተ/ቁ	ጥያቄ	ምላሽ/መልስ	ኮድ	ማለፍ/ መዝለል
86	ሁነኛ በሚባል ቀን ለምን ያህል ሰአት ቁጭ ወይም ጋደም ይላሉ?	ሰአት-----------------------<br>ደቂቃ----------------	P18 (a-b)	
**ሌሎች ባህሪያትን በተመለከተ**				
**ቁልፍ፤ የደም ግፊት በሽታን በተመለከተ**				
ተ/ቁ	ጥያቄ	ምላሽ/ መልስ	ኮድ	ማለፍ/ መዝለል
87	የደም ግፊት መጠንዎን በጤና ሙያተኞች ተለክተው ያውቃሉ?	አዎ-------------------1<br>የለም-----------------2	H15	የለም → ተ/ቁ 93

88	የደም ግፊት መጠንዎን ከፍ እንዳለ በጤና ሙያተኞች ተነግሮዎት ያውቃል?	አዎ-------------------1<br>የለም-----------------2	H16a	የለም → ተ/ቁ 93
89	ባለፉት 12 ወራት የደም ግፊት መጠንዎ ከፍ ብሏል ተብለው ያውቃሉ?	አዎ--------------------1<br>የለም------------------2	H16b	
90	በለፉት ሁለት ሳምንታት በሀኪም የታዘዘ የደም ግፊት መድሀኒት ወስደዋል?	አዎ---------------------------1<br>የለም--------------------------2	H17	
91	የደም ግፊት ችግርዎን በተመለከተ የባህል መድሀኒት አዋቂ ጋራ ራስዎን አሳይተው ነበር?	አዎ---------------------------1<br>የለም--------------------------2	H18	
92	አሁን ባሉበት ሁኔታ የደም ግፊት ችግርዎን በተመለከተ ያስተካክላል ብለው የሚያምኑትን የባህል መድሀኒት እየወሰዱ ነው?	አዎ---------------------------1<br>የለም--------------------------2	H19	
93	በቤተሰብዎ ውስጥ የደም ግፊት በሽታ ያለበት ሰው ነበር(አለ)?	ቤተሰብ--------------------------1<br>አባት----------------------------2<br>እናት----------------------------3<br>ወንድምና እህት----------------4<br>ልጆች--------------------------5<br>ማንም የለም--------------------6<br>አላውቅም---------------------88	H20	
**ቁልፍ፤ የስኳር በሽታን በተመለከተ**				
**ተ/ቁ**	**ጥያቄ**	**ምላሽ/መልስ**	**ኮድ**	**ማለፍ/ መዝለል**
94	በደምዎ ውስጥ ያለው የስኳር መጠን ተለክቶ ያውቃል?	አዎ--------------1<br>የለም-----------2	H21	የለም → ተ/ቁ 100
95	በደምዎ ውስጥ ያለው የስኳር መጠን በጤና ሙያተኛ ተለክቶ ከፍተኛ ነው ወይም የስኳር በሽታ አለበዎት ተብለው ያውቃሉ?	አዎ--------------1<br>የለም-----------2	H22	የለም → ተ/ቁ 100
96	ባለፉት 12 ወራት በደምዎ ውስጥ ያለው የስኳር መጠን ከፍተኛ ነው ወይም የስኳር በሽታ አለበዎት ተብሎ ተነገሮዎታል?	አዎ--------------1<br>የለም------------2<br>አላስታውስም------88	H23	
97	በለፉት ሁለት ሳምንታት በጤና ሙያተኞች የታዘዘ የስኳር በሽታ መድሀኒት እየወሰዱ ቆይተዋል?	አዎ--------------1<br>የለም-----------2	H24	
98	በደምዎ ውስጥ ያለው የስኳር መጠን በመጨመሩ ወይም የስኳር በሽታዎን አስመልክቶ ወደ ባህላዊ መድሀኒት አዋቂ ዘንድ ሄደው ነበር?	አዎ--------------1<br>የለም-----------2	H25	
99	በአሁኑ ወቅት በደምዎ ውስጥ ያለው የስኳር መጠን በመጨመሩ ወይም የስኳር በሽታዎን ለማስታገስ የባህል መድሀኒት እየወሰዱ ነው?	አዎ--------------1<br>የለም-----------2	H26	

የመጠይቅ መለያ ኮድ

100	በቤተሰብዎ ውስጥ የስኳር በሽታ ያለበት ሰው ነበር(አለ)?	ቤተሰብ----------------1<br>አባት-------------------2<br>እናት--------------------3<br>ወንድምና እህት--------4<br>ልጆች--------------------5<br>ማንም የለም------------6<br>አላውቅም-------------88	H27	
**ቁልፍ፤ በደም ውስጥ የኮሌስትሮል መጠን መጨመርን በሚመለከት**				
**ተ/ቁ**	**ጥያቄ**	**ምላሽ/መልስ**	**ኮድ**	**ማለፍ/ መዝለል**
101	በደም ውስጥ ያለውን የኮሌስትሮል ቅባት መጠን በጤና ሙያተኛ ተለክተው ያውቃሉ?	አዎ---------------1<br>የለም-------------2	H28	የለም<br>→<br>ተ/ቁ 107
102	በጤና ሙያተኛ በደምዎ ውስጥ ያለው የኮሌስትሮል ቅባት መጠን ከፍተኛ ነው ተብለው ያውቃሉ?	አዎ---------------1<br>የለም-------------2	H29	የለም<br>→<br>ተ/ቁ 107
103	ባለፉት 12 ወራት በጤና ሙያተኛ በደምዎ ውስጥ ያለው የኮሌስትሮል ቅባት መጠን ከፍተኛ ነው ተብለዋል?	አዎ---------------1<br>የለም-------------2	H30	
104	ባለፉት ሁለት ሳምንታት በጤና ሙያተኛ በደምዎ ውስጥ ያለውን ከፍተኛ የኮሌስትሮል ቅባት መጠን የሚያስተካክል በአፍ የሚወሰድ መድሃኒት ወስደዋል?	አዎ---------------1<br>የለም-------------2	H31	
105	በደምዎ ውስጥ ያለውን ከፍተኛ የኮሌስትሮል ቅባት መጠን ተከትሎ የባህላዊ መድሀኒት አዋቂ ዘንድ ሄደው ነበር?	አዎ---------------1<br>የለም-------------2	H32	
106	በአሁኑ ወቅት በደምዎ ውስጥ ያለውን ከፍተኛ የኮሌስትሮል ቅባት መጠን ተከትሎ የባህላዊ መድሀኒት የሚወስዱት አለ?	አዎ---------------1<br>የለም-------------2	H33	
107	በቤተሰብዎ ውስጥ ከፍተኛ የኮሌስትሮል ቅባት መጠን ያለበት ሰው ነበር(አለ)?	ቤተሰብ---------------1<br>አባት------------------2<br>እናት------------------3<br>ወንድምና እህት-------4<br>ልጆች------------------5<br>ማንም የለ-------------6<br>አላውቅም------------88	H34	
**ክፍል ሁለት፤ አካላዊ ልኬቶች**				
**ቁልፍ፤ የደም ግፊት መጠን**				
**ተ/ቁ**	**ጥያቄ**	**ምላሽ/መልስ**	**ኮድ**	**ማለፍ/ መዝለል**
108	የጠያቂው ስምና መለያ ቁጥር	---------------	M1	
109	የደም ግፊት መለኪያ መሣሪያው ቁጥር	----------	M2	

የመጠይቅ መለያ ኮድ

110	የተጠቀሙበት ካፍ/cuff/ መጠን	ትንሹ---------------1<br>መጠነኛ------------2<br>ትልቁ--------------3	M3	
111	ንባብ 1	ሲስቶሊክ------------------------<br>ሚሊ ሜትር ሜረኩሪ<br>ዲያስቶሊክ----------------------<br>ሚሊ ሜትር ሜረኩሪ	M4a<br>M4b	
112	ንባብ 2	ሲስቶሊክ------------------------<br>ሚሊ ሜትር ሜረኩሪ<br>ዲያስቶሊክ ----------------------<br>ሚሊ ሜትር ሜረኩሪ	M5a<br>M5b	
113	ንባብ 3	ሲስቶሊክ-------------------------<br>ሚሊ ሜትር ሜረኩሪ<br>ዲያስቶሊክ----------------------<br>ሚሊ ሜትር ሜረኩሪ	M6a<br>M6b	
114	ባለፉት ሁለት ሳምንታት የደም ግፊት መጠንዎ ስለ ጨመረ ከሃኪም የደም ግፊት መድሃኒት እንዲወስዱ ተመክረው ነበር?	አዎ---------------1<br>የለም-------------2	M7	
**ቁልፍ፤ ቁመትናክብደት**				
115	የጠያቂው መለያ ቁጥር	------------------------	M8	
116	የቁመት እና ክብደት መጠንን ለመለካት የተጠቀሙበት መሣሪያ ቁጥር	የቁመት--------------------<br>የክብደት------------------	M9	
117	ቁመት	---------------ሴ.ሜ	M10	
118	ክብደት<br>የክብደት መጠናቸው ከልኬት በላይ ከሆነ 666.6 ብለው ይመዝግቡ	------------------ኪ.ግ	M11	
**ቁልፍ ፤ የወገብ ዙሪያ ልኬት**				
119	የወገብ ዙሪያ ለመለካት የተቀሙበት መሣሪያ መለያ ቁጥር	------------------	M12	
120	የወገብ ዙሪያ ልኬት	--------------------ሴ.ሜ	M13	
**የተብራራ ምላሽ፤ የዳሌ ዙሪያ ልኬት እና የልብ ምት መጠን**				
121	የዳሌ ዙሪያ ልኬት	-------------ሴ.ሜ	M14	
122	የልብ ምት መጠን			
	ንባብ1	-------------በደቂቃ	M15a	
	ንባብ2	------------በደቂቃ	M15b	
	ንባብ 3	------------በደቂቃ	M15c	
**ክፍል 3፤ በደም ውስጥ ያሉማዕድናት/ ኬሚካል መጠን**				
**ቁልፍ፤ በደም ውስጥ ያለ የስኳር መጠን**				

123	ባለፉት 12 ሰዓታት ውሃን ሳይጨምር የበሉት ወይም የጠጡት ነገር አለ?	አዎ---------------1<br>የለም-------------2	B1	
124	የቴክኒክ ባለሙያው መለያ ቁጥር	----------------------	B2	
125	የተጠቀሙበት መሣሪያ መለያ ቁጥር	----------------------	B3	
126	ደም የወሰዱበት ዕለታዊ ሰዓት	ሰአት----------ደቂቃ----------	B4	
127	ምግብ ሳይመገቡ በፊት በደም ውስጥ የነበረው የስኳር መጠን<br>(በሚሊሞል ወይም በ ሚግ/ዴሊ)	------------ሚሊሞል/ሊ<br>-------------ሚግ/ዴሊ	B5	
128	በዛሬው ዕለት የደም ውስጥ የስኳር መጠንዎ ከፍ ከማለቱ ጋር በተያያዘ በሃኪም የታዘዘለዎትን ኢንሱሊን ወይም ሌላ መድሃኒት ወስደዋል?	አዎ--------------------1<br>የለም------------------2	B6	
**ቁልፍ፤ በደም ውስጥ ያለውን የቅባት መጠን**				
129	የመለኪያ መሣሪያው መለያ ቁጥር	--------------------	B7	
130	የተሳታፊው መለያ ቁጥር	--------------------	B8	
131	የተሳታፊው መኖሪያ ቦታ	ክፍለ ከተማ--------------------<br>ወረዳ--------------------	B9	
132	የናሙናው መለያ ቁጥር	--------------------	B10	
133	በደም ውስጥ ያለው አጠቃላይ የኮሌስትሮል መጠን (በሚሊሞል ወይም በ ሚግ/ዴሊ)	------------------ሚሊሞል/ሊ<br>------------------ሚግ/ዴሊ	B11	
134	ባለፉት ሁለት ሳምንታት ውስጥ በደምዎ ውስጥ ያለው የኮሌስትሮል ቅባት መጠን በመጨመሩ ምክንያት በሃኪም የታዘዙለዎት መድሃኒቶች ተጠቅመው ነበር?	አዎ-------------------1<br>የለም-----------------2	B12	
**የተብራራ ምላሽ፤ ትራይግላይሴራይድ እና ከፍተኛ ዴንሲቲ ያለው የኮለስትሮል ቅባት ልኬቶች**				
135	የትራይግላይሴራይድ መጠን<br>(በሚሊሞል ወይም በ ሚግ/ዴሊ)	----------------ሚሊሞል/ሊ<br>----------------ሚግ/ዴሊ	B13	
136	ከፍተኛ ዴንሲቲ ያለው የኮለስትሮል ቅባት መጠን (በሚሊሞል ወይም በ ሚግ/ዴሊ)	----------------ሚሊሞል/ሊ<br>------------------ሚግ/ዴሊ	B14	

የመጠይቅ መለያ ኮድ

