# Supplementary material for: Prevalence and risk factors of hypertension among adults: A community based study in Addis Ababa, Ethiopia
Source: PLoS One. 2021 Apr 1;16(4):e0248934. doi: 10.1371/journal.pone.0248934 (PMC8016337; doi:10.1371/journal.pone.0248934)
Supplement: Attachment — Submitted filename: Manuscript ID number.docx [file pone.0248934.s004.docx]

Manuscript ID number:

PONE-D-20-26679

Title of paper:

Prevalence and risk factors of hypertension among adults: a Community Based Study in Addis Ababa, Ethiopia

**Evaluation**

Despite careful approach to investigate Prevalence and risk factors of hypertension, manuscript needs minor revisions to make it easy to understand before being published.

**General comments:**

1. Language editing **strongly recommended**
2. The body of the text suffers from several spelling and grammatical errors. Please consider a professional language edit. Example: scare (page 3 first paragraph),
3. Standardized your tables by removing the boarders and include P values in table 3

**Page 2**

4. In the abstract result section, almost all (96.2%) of participants consume vegetables and or fruits less than five times per day.

**Is that feasible consuming vegetable & fruits five times per day in Ethiopian context? Or you mean five times per week? Make it clear**

**Page 3 & 4**

5. Moreover, in Ethiopia non-communicable diseases such as hypertension and diabetes mellitus appear on the list of leading causes of morbidity and mortality in the hospitals and regional health bureaus across the country. A report by Ethiopian Public Health Institute (EPHI) in 2016 showed that 95% of the Ethiopian adult populations have 1-2 Non-Communicable Diseases risk factors (6, 7). But, there were scare data with regard to the magnitude and risk factors of common non communicable disease at the community level in Ethiopia including the study area Addis Ababa. Moreover, the study area represents the largest urban center in Ethiopia, hosting about 25% of the urban population in the country (5).

**Since you are not intended to study all types of non-communicable diseases better to focus on hypertension). Paragraph 4, page 3 needs both language & grammatical edition.**

**Page 5**

6. The method section, selection of the study participant,

**the last paragraph a total of 3724 all needs to reconsider again**

**page 4**

7. A community based cross-sectional study was conducted from June to October 2018 in Addis.

**Please provide more precise date of study begin and termination**

**Page 5**

8. Multi-stage cluster sampling techniques was employed. Seven of the ten sub-cities were selected purposefully by considering the area that was found, the population density and the economic activities.

**You didn’t say anything about how you determine the sample size. How you calculate your sample size, what assumptions you used to calculate your sample size both for the magnitude & factors. Also, important you should show us how you allocate the number of participants to Sub-cities or Woreda Or Kebeles, Ketenas & households?**

**Page 5**

9. One of the methods of maintaining the quality of data is keeping the data collection instrument valid & reliable (in you case weight scale & BP apparatus, the STEPS Questionnaire). In this regard you didn’t say anything.

**How you maintain the reliability & validity of this instruments? We need more clarification on this issue**

**Page 9**

10. In the description of the study participants, result section, you calculate both the mean with SD and Median with IQR for the respondents’ age.

**What was the reason and which one was appropriate for your data? Need clarification**

**Page 11**

11. In **Tobacco use section to told us** about 4.2% (150), of the survey participants were current smokers (daily and non-daily smokers) again in the last two sentence of the same section you presented, fifty-five (1.61%) were ever smoked cigarettes and One hundred nineteen (3.4%) were passive smoking or second-hand smoke.

**What does this imply? Are these 55 peoples being among 150 who currently smoke? Needs to be clarified.**

**Page 13**

12. Weight and height measurement were taken from all participants 3560 and the BMI was calculated for those participants. But you didn’t show how you calculate the BMI (only you defined BMI in the operational definition).

**It is important to show how was the BMI calculated in the methods section. The procedure you used needs to be clearly kept in the method section**

**Page 13**

13. You told us that blood sample was collected from 20% of the total study participants.

**It is not sufficient to write 20% of total you need to write the actual number of participants you collect blood sample.**

**Page 13**

14. In the result section, prevalence of hypertensin, you presented the overall prevalence of hypertension was 29.24% (95% CI: 27.75-30.74), slightly higher among men 30.13 (95% CI: 27.82-32.44), than women 28.58 (95% CI: 26.66-30.54) even though the difference was not statistically significant (χ^2^=1.015, P= 0.314).

**But in the factors associated with you stated that sex had significant association with hypertension (The odds of hypertension was almost two times higher AOR= 1.88 (95% CI: 1.18-2.99) among males as compared to females). Needs clarification and reconsideration.**

**Page 19 discussion section**

15. Hypertension is an important modifiable risk factor for cardiovascular disease (CVD). It currently accounts for about 13.5% of annual global deaths. Hypertension is directly responsible for 54% of all strokes and 47% of all coronary heart disease worldwide. Moreover, over half of this burden occurs in individuals aged 45–69 years, which is the most productive segment of the population (31).

**Better to start your discussion by summary of your results and good if you use this in the introduction section**

Page 19

16. …………. So, the prevalence of hypertension in the current study is slightly higher among men than women which is comparable with a community based study conducted in Addis Ababa, Ethiopia which reported the prevalence of hypertension was 31.5% and 28.9% among males and females, respectively (5). Moreover, this study is also comparable with other community-based studies conducted in Jalalabad, Afghanistan (28.4), Kenya (29.4%), Uganda 375 (30.5%), and Gondar city (28.3%) (12-15).

**Here first you talk about the association between hypertension and gender or sex and on the next paragraph back to compare the prevalence with other studies. I see some confusion here I think you would want to change the order of the paragraph?**

**Page 20**

17. ……… which the risk of hypertension increases with age. This is mainly due to systolic blood pressure increase with age, mainly because of reduced elasticity (increased stiffness) of the large conduit arteries (26). In this study respondents aged 30-49 years; had 3 times higher risk of hypertension and even moreover, it is 8 times higher risk among participants aged 50 years and above.

**What is your message here for the patients and health care providers you provide? Is there anything that recommend to tackle this problem or age? You should better to emphasize on modifiable factors than non-modifiable like age & sex. Need your consideration**

Page 20,

18. This finding (especially obese category) was in line with previous reports from Ethiopia, Kenya, Uganda, Sudan, Bangladesh, and Cameroon (13, 15, 17, 18, 21, 22, 24, 25, 27). Moreover, the risk of hypertension was 2 times higher among abdominally obese respondents and this finding is in line with other studies (24, 25, 28, 29) and the same to the level of triglyceride also.

**Since this is the most important area that your recommendation is focused, comparing the findings is not sufficient. Better to find the reason of similarity or differences and give your recommendation or message based on that. Therefore, you need to work on it and put your recommendation.**

**Page 21, first paragraph**

19. In contradiction, in this study the above variables were not significantly associated with hypertension. The inconsistency of these findings may be due to the low prevalence of these factors in the community especially among females.

**What does it mean? I don’t think your reason for differences is correct. May you need to find tangible reason for this difference.**

**Page 21**

20. Additionally, the respondents might not know whether they had a family history of hypertension or diabetes due to the silent killer nature of the diseases this may underestimate the prevalence of the diseases.

**How the silent killer nature of the disease affects the prevalence of hypertension since the prevalence was determined by measuring their blood pressure? Or you want to say the severity of the disease? Not clear**

**Do you think diabetes is a silent killer? Since your objectives did not include diabetes why you include here?**

**Page 21**

21. The other reason should since some of the information was based on self-report and is subjected to social desirability and recall biases.

**These issues are very critical in research. How you manage this social desirability and recall biases since this can affect severely your findings? You have to show us either in the discussion or method section how you control theses biases clearly?** In addition, with all these short comings or limitations do think your research could be eligible for publication? Better to avoid those limitations that can be controlled methodologically

**Page 21**

22. In the conclusion section …. There was a high prevalence of hypertension among adults in the Addis Ababa city and this may show a hidden epidemic in the population. What is your reference to say high prevalence or to conclude this is a hidden epidemic? You have to show here
